# The Challenging Road to Hepatitis C Virus Eradication

**DOI:** 10.3390/jcm10040611

**Published:** 2021-02-05

**Authors:** Isidoro Martínez, Pablo Ryan, Jorge Valencia, Salvador Resino

**Affiliations:** 1 Unidad de Infección Viral e Inmunidad, Centro Nacional de Microbiología, Instituto de Salud Carlos III, 28220 Majadahonda, Spain; 2 Hospital Universitario Infanta Leonor, 28031 Madrid, Spain; pabloryan@gmail.com (P.R.); jorge_vlr@yahoo.es (J.V.); 3 Unidad de Reducción de Daños “SMASD”, 28004 Madrid, Spain

Hepatitis C virus (HCV) infection remains a substantial health problem as a leading cause of chronic liver disease worldwide [1]. Chronic hepatitis C (CHC) develops in 75–100% of patients who remain plasma HCV-RNA positive after acute hepatitis C. The World Health Organization (WHO) has estimated that there are about 71 million individuals with CHC worldwide, many of whom are unaware of their infections [1]. CHC causes persistent liver inflammation, leading to cirrhosis development in approximately 10–20% of patients after 20–30 years of HCV infection. Cirrhotic patients have a 1–5% annual risk of developing hepatocellular carcinoma and a 3–6% risk of hepatic decompensation. Following an episode of decompensation, the risk of death in the following year is between 15 and 20% [2].

HCV prevalence is increased in marginalized populations, mostly among people who inject drugs (PWIDs). Currently, the burden of active hepatitis C in PWIDs remains high [3], with a worldwide HCV prevalence of around 39%, representing an estimated 6.1 million people (9% of all global infections) [4]. Additionally, the rates of HCV infection, transmission, and reinfection are augmented in this group due to ongoing risk behaviors despite needle and syringe exchange programs [5,6]. Additionally, the percentage of PWIDs undiagnosed with hepatitis C is significant since they tend to have limited access to health systems.

A vaccine for HCV is currently not available, but, after more than a decade in which interferon (IFN) and ribavirin were the mainstays of anti-HCV treatment, the licensure of direct-acting antiviral agents (DAAs), which block HCV replication, has revolutionized HCV therapy. IFN-free therapy with DAAs has resulted in a sustained virological response (SVR) in almost all treated patients (>95%), even in PWIDs and patients coinfected by human immunodeficiency virus (HIV) [7]. Due to its high efficacy, low toxicity, and good tolerability, it has been possible to treat and cure, in the last five years, most HCV patients who have been followed at the clinic, reducing the volume of patients who are currently attending. The WHO aims to reduce new HCV infections and deaths by 2030 [8]. DAAs and novel HCV screening are the two tools that have led the WHO to declare a global war against HCV. The “Global Health Sector Strategy on Viral Hepatitis, 2016–2021” proposed reducing new viral hepatitis infections by 90% and reducing viral hepatitis deaths by 65% in 2030 [8]. 

Elimination efforts and responses from healthcare institutions and public health have been robust in most countries, and restrictions due to the high cost of the DAAs have progressively been eliminated. However, there is still a high proportion of persons with high-risk behaviors who have active HCV infections and have not been diagnosed and treated [9]. Additionally, the risk of reinfection among these people is very high [5]. This population group includes drug users, the homeless, and people with psychiatric illnesses, among others [10]. Therefore, the HCV elimination strategy requires reducing risky behaviors, expanding HCV testing, and unrestricted access to treatment [7]. Proactive HCV screening from frontline healthcare personnel and early HCV treatment from hepatologists and infectious disease physicians will be crucial. However, access to HCV therapy and decentralized treatment is a challenge.

Although there are no restrictions in most Western healthcare systems, excluded populations have limitations in accessing healthcare and HCV therapy. The proactive search of these populations has required a network of different health and non-health professionals in an interdisciplinary approach. Moreover, the lack of access to treatment is due to multiple personal and system barriers. Alternative and parallel circuits may be used in these populations to link vulnerable persons to care. HCV care must be changed from a traditional clinical practice to a clinical, social, and public health approach.

HCV screening is cost-effective since the early identification of HCV infection allows patients to benefit from DAA therapy, limits HCV transmission, and avoids clinical procedures and interventions in advanced liver disease stages [11]. In this context, the early diagnosis of HCV infection and treatment is crucial for eradicating the virus in PWIDs [4]. Thus, HCV infection prevalence can be substantially reduced through significant increases in the frequency of diagnosis and access to HCV treatment in target populations [4]. The most pragmatic approach to implementing HCV elimination would be to break down elimination goals into smaller goals for population segments (microelimination) [10]. Thus, diagnosis, treatment, and prevention interventions could be delivered more quickly and efficiently using innovative methods [12]. This strategy could be useful for marginalized populations with high HCV prevalence, such as PWIDs [13].

The diagnosis of HCV infection is performed mainly through specialized health centers using serological tests, followed by a confirmatory test for active hepatitis C (positive HCV RNA). This standard strategy requires multiple visits to the hospital, which leads to the loss of patients during follow-up, mainly in the infected PWID population [6,14]. Thus, hepatitis C testing and diagnosis remain inadequate in this group, in terms of both numbers and procedures, because PWIDs often do not access health services or are not referred to the hospital [7]. Besides, PWIDs have lower rates of linkage to care, particularly hospital care and HCV treatment initiation, due to their dependence on drug use, psychiatric illnesses, and social and economic problems [15,16]. 

HCV screening at the point of care (PoC) increases test acceptance and the linkage to care in PWIDs [17]. HCV screening via finger-stick dried blood spot (DBS) samples is inexpensive and straightforward and can be implemented with minimal training [7]. It obviates the need for venipuncture and syringes, requires only minimal amounts of blood, and needs little storage space. Consequently, the samples are easy to carry and handle. Besides, several systematic reviews have shown that HCV screening with DBS has good diagnostic performance [18] and enhances HCV testing in PWIDs [19], who accept this method more widely than venous extraction. Still, the linkage to care is low in PWIDs because only around 50% of HCV-infected people start HCV treatment [15,16]. 

Alternative PoC strategies are being implemented to improve HCV diagnosis and linkage to care in a single visit (test-and-treat approach) [20]. Thus, a quick serological test followed by a rapid test for HCV RNA or core antigen is successfully used. This strategy reduces the time from sample collection to HCV diagnosis, and improves the acceptability of testing and linkage to treatment [7,21]. Another approach to improving the linkage to care is the decentralization of HCV treatment, namely, providing it through physicians who deliver therapy at the PoC or remove prescribing restrictions for DAAs outside of the hospital setting [15,16]. The incorporation of peer navigators, linkage-to-care coordinators, and mobile medical units also reinforces the linkage to care [15,20].

Besides HCV screening and linkage to care, additional problems challenge successful treatment, such as the emergence of resistance-associated substitutions (RAS) in patients who do not achieve an SVR. HCV shows enormous genetic diversity, and a single mutation may be sufficient to confer antiviral resistance to DAAs, with a low barrier to resistance [22]. Despite the high SVR rates in PWIDs, HCV strains with established RAS in individuals from this population failing DAA treatment can be easily transmitted to others by sharing injecting equipment [23]. Moreover, some HCV genotypes display an inherent resistance to certain DAAs [22], which may increase their frequency in PWIDs, a niche suitable for expanding RAS. Therefore, it is essential to have strategies to monitor the appearance of HCV strains resistant to DAAs.

In conclusion, given the lack of an effective vaccine, extensive HCV screening and treatment are indispensable for eradicating HCV. However, achieving this goal is challenging, and efforts should focus on marginalized populations due to the high prevalence of infection, risk behaviors, and poor linkage to care. 

## Abbreviations

HCV	Hepatitis C virus
CHC	Chronic hepatitis C
WHO	World Health Organization
PWIDs	People who inject drugs
IFN	Interferon
DAAs	Direct-acting antivirals
SVR	Sustained virological response
HIV	Human immunodeficiency virus
PoC	Point of care
DBS	Dried blood spot
RAS	Resistance-associated substitutions
